# Dietary Tea Polyphenols Alleviate Acute-Heat-Stress-Induced Death of Hybrid Crucian Carp HCC2: Involvement of Modified Lipid Metabolisms in Liver

**DOI:** 10.3390/metabo15040229

**Published:** 2025-03-27

**Authors:** Na Zhang, Jinsheng Tao, Qifang Yu, Gege Sun, Xiaopeng Liu, Weirong Tang, Lina Zhang, Zhe Yang

**Affiliations:** 1 State Key Laboratory of Developmental Biology of Freshwater Fish, Hunan Normal University, Changsha 410081, China; biologyzhang@aliyun.com (N.Z.); yuqf@hunnu.edu.cn (Q.Y.); xpliu@hunau.edu.cn (X.L.); 2 Engineering Research Center of Polyploid Fish Reproduction and Breeding of the State Education Ministry, College of Life Sciences, Hunan Normal University, Changsha 410081, China

**Keywords:** tea polyphenols, crucian carp, heat stress, metabolomic, lipid metabolism

## Abstract

Background: Global warming poses significant challenges to aquaculture, as elevated water temperatures adversely affect fish health and survival. This study investigated the effects and potential mechanisms of dietary tea polyphenols (TPs) on acute heat stress and survival in hybrid crucian carp HCC2. Methods: The fish in the control (CON) group and heat stress group (HS group, three replicates, each containing 20 fish, *n* = 60 per group) were fed diets with 0 mg/kg TPs, and the three experimental groups (HSLTP, HSMTP, and HSHTP, *n* = 20 × 3 replicates) were fed the diets with 100, 200, or 400 mg/kg TPs for 60 days. Further, fish in the experimental groups (HS, HSLTP, HSMTP, and HSHTP) were exposed at 38 °C for 24 h to induce acute heat stress. Survival data and serum and tissue samples were collected for the analysis. Metabolomics using UPLC-Q-TOF/MS was employed to evaluate the metabolite changes in the fish livers. Results: Notably, dietary TPs significantly improved survival rates and antioxidant enzyme levels and reduced serum ALT, AST, cortisol, glucose, MDA, and liver HSP-70 levels in the heat-stressed fish. Metabolomic analysis revealed that TPs modulated lipid metabolism, particularly glycerophospholipid and arachidonic acid pathways, which may contribute to a higher tolerance to acute heat stress. Conclusions: These findings suggest that TPs are a promising, eco-friendly feed additive for protecting fish from heat stress and optimizing aquaculture practices.

## 1. Introduction

A persistent and global concern is the increase in global water temperature caused by the greenhouse effect [1]. Water temperature is a critical environmental factor in aquaculture, and fluctuations in heat on a daily or seasonal basis, as well as changes in culture water temperature, have a significant impact on fish growth [2]. Fish frequently encounter acute heat stress, especially during transport and seasonal changes. These conditions can adversely affect their immune system, antioxidant capacity, muscle mass, and growth performance due to the abrupt changes in their external environment [3]. For instance, high environmental temperatures (36 °C) generally enhance lipid metabolism, diminish fatty acid synthesis, and disrupt the immune system in grass carp [4].

Hybrid fish are an indispensable part of fish species, which show the advantages of a fast growth rate, high immunity, strong ecological adaptability, and strong transportation tolerance [5]. In recent years, we initially cultivated a new type of high-quality hybrid crucian carp [(*Carassius auratus cuvieri*(♀) × *Carassius auratus red variety*(♂)) × *Carassius auratus cuvieri*(♂)], namely “Hefang crucian carp II” (HCC2) by applying biotechnology techniques combining population breeding and sexual hybridization. HCC2 fish have advantages such as rapid growth, a high fertilization rate, a high hatching rate, a high protein content, and a high content of flavor amino acids [6]. The growth performance of crucian carp peaks when water temperatures range from 25 to 28 °C. However, as water temperatures increase to between 28 and 31 °C, the relative growth rate markedly decelerates, accompanied by a reduction in lymphocyte counts and an elevation in neutrophil levels [7]. Unfortunately, the summer water temperatures in the subtropical regions where crucian carp are widely distributed often exceed 28 °C [8], which poses a significant challenge to crucian carp being able to fully exhibit their inherent growth advantages. It is necessary to find effective ways to solve the heat-stress-caused problems during HCC2 production.

Plant extracts, rich in bioactive ingredients, are known as eco-friendly natural and functional additives. Natural and functional additive supplementation has been recommended as a useful method to mitigate fish stress [9]. TPs exhibit a diverse array of pharmacological and biological activities, including antioxidant [10], anti-inflammatory [11], and antimicrobial activities [12] and intestinal microbe regulation [13]. The sustainability and cost-effectiveness of TPs serve as significant advantages when they are utilized as feed additives for farm animals. TPs could partially substitute for synthetic antioxidants (such as butylated hydroxytoluene and butylated hydroxyanisole) and antibiotics, thereby significantly reducing the environmental pollution caused by chemical residues in feed to soil and water bodies [14]. Furthermore, advancements in industrial extraction methods for tea polyphenols, including membrane separation and supercritical CO_2_ extraction, have led to a significant reduction in production costs, which are approaching those of synthetic antioxidants [15].

Recently, TPs have garnered significant attention due to their anti-stress properties [16]. Nuclear factor erythroid 2-related factor 2 (Nrf2) and nuclear factor kappa B (NF-κB) play crucial roles in cellular protection and inflammatory responses. (-)-Epigallocatechin-3-gallate (EGCG), a bioactive compound found in TPs, can modulate these signaling pathways, thereby enhancing the antioxidant status of heat-stressed poultry [17]. Similarly, TPs can protect bovine intestinal epithelial cells from HS-induced adverse effects by alleviating oxidative stress and inflammatory responses [18]. Moreover, TP supplementation has been recommended as a useful method to mitigate fish stress. Dietary supplementation with TPs alleviated ammonia-induced oxidative damage in juvenile Wuchang bream (*Megalobrama amblycephala*) by enhancing their antioxidant and immune capacities [19]. The incorporation of TPs into oxidized fish oil-based feed enhances intestinal morphology, enriches beneficial microbiota, and optimizes hepatic nutrient metabolism in spotted sea bass (*Lateolabrax maculatus*) [20]. Further, Li et al. found that under heat-stress conditions, TPs could alleviate the stress response of Nile tilapia [21]. However, the literature on the effects of TPs in mitigating heat stress in fish is limited, and the mechanisms by which TPs enhance the health status of aquatic animals remain underexplored. The metabolomics methodology that has recently emerged in the field of aquaculture holds the potential to be a breakthrough [22].

In the present study, we hypothesize that TP supplementation in feed has the potential to alleviate heat stress in HCC2. To our knowledge, this is the first study to integrate UPLCQTOF/MS-based metabolomics with physiological indices to elucidate how TPs modulate lipid metabolism pathways in fish under acute heat stress, providing a mechanistic foundation for TPs’ application in aquaculture.

## 2. Materials and Methods

### 2.1. TPs and Experimental Diets

Based on the nutritional requirements of crucian carp, the compositions of the five experimental diets in this study are listed in Table 1. Four isonitrogenous and isolipidic diets were formulated, containing 0, 100, 200, and 400 mg/kg of TPs, respectively. TPs derived from green tea (purity > 98%) were provided by the National Research Center of Engineering Technology for Utilization of Functional Ingredients from Botanicals, Hunan Agricultural University, Changsha, China. The main components of TPs are shown in Supplementary Table S1.

The main ingredients were fully ground and filtered through an 80-mesh sieve. Subsequently, the feedstuffs were mixed with water for homogeneity and formed into extruded floating pellets (particle diameter: 1 mm) using a small-scale extruder (EF-003, Zhuhai Huolibao Feed Co., Ltd., Zhuhai, China). Eventually, all the experimental diets were airdried at 65 °C and conserved at −20 °C until they were used.

### 2.2. Fish and Experimental Conditions

HCC2 with average initial weights of 2.05 ± 0.03 g were obtained from the State Key Laboratory of Developmental Biology of Freshwater Fish, Hunan Normal University (Changsha, China). The fish were F2-generation lab-raised HCC2, minimizing wild-caught stress variability. The fish were acclimated under natural conditions (26.0 ± 2 °C) with a natural light–dark cycle for one week. After acclimation, the 1500 fish were randomly divided into five groups, with three replicates, of 100 fish in a 2 m^3^ cage for 60 days. During the trial, the fish in the control (CON) group and heat stress (HS) group were fed diets with 0 mg/kg TPs and the three treatment groups (HSLTP, HSMTP, and HSHTP) were fed diets with 100, 200, or 400 mg/kg TPs. Water quality parameters were monitored daily, and no significant changes were observed in dissolved oxygen (6.49–6.82 mg/L), temperature (26.0 ± 2 °C), pH (7.16–7.38), or un-ionized ammonia nitrogen (<0.001 mg N/L).

Upon the completion of the trial, 20 fish were randomly selected from each of the three replicate cages, resulting in a total sample size of 60 fish per experimental group. These fish were subsequently transferred to a single 100 L experimental tank that was equipped with a temperature control system.

### 2.3. Heat Stress Experiment and Sample Collection

Based on several pre-experiments conducted in our laboratory, we established a control group (CON), maintained at 26 °C, and four high-temperature groups (HS, HSLTP, HSMTP, and HSHTP), exposed to 38 °C. The water temperature was controlled by electrical heaters, which gradually increased by 1 °C/30 min. This gradual temperature increase aligned with protocols used in prior studies on crucian carp thermal tolerance [23]. The fish were put into all the tanks at a water temperature of 26 °C, thereby initiating the heat-stress experiment. The CON group (26 °C) was maintained under the same experimental duration (24 h) as the heat-stressed groups to control for time-dependent effects. The four high-temperature groups subsequently reached a peak temperature of 38 °C after 6 h. Throughout the experiment, adequate dissolved oxygen was maintained, and fish behavior and survival rates (SRs) were monitored and recorded.

After the 24 h heat-stress induction, 6 survival fish were randomly removed from each treatment. The fish were anesthetized using MS-222 (Merck, Darmstadt, Germany). Blood was collected from the caudal vein and immediately centrifuged at 3000× *g* for 10 min at 4 °C. In each replicate tank, the serum from three fish was pooled and mixed equally (resulting in a total of six composite serum samples per group). The separated serum was then frozen and stored at −20 °C for subsequent analysis. Subsequently, multiple samples from the most distal portion of the posterior lobe of the liver were rapidly excised and immediately flash-frozen using liquid nitrogen.

### 2.4. Stress Indicators

The levels of alanine transaminase (ALT), aspartate aminotransferase (AST), glucose, and total bilirubin (T-BIL) in the serum were monitored by the employed assay kits, manufactured by Nanjing Jiancheng Bioengineering Institute in China. The concentrations of cortisol in the serum and heat shock protein-70 (HSP-70) in the liver were detected using a fish-specific enzyme-linked immunosorbent assay (ELISA) kit (Cusabio, Wuhan, China).

### 2.5. Antioxidant Capacity

The frozen liver samples were thawed at 4 °C, after which 100 mg of the livers were transferred to 1.5 mL microcentrifuge tubes. Each tube was then supplemented with three grinding beads and 1 mL of extraction solution. Liver tissue was homogenized with 3 mm zirconia beads at 6000 rpm for 60 s (Tissuelyser LT, Qiagen, Shanghai, China). The antioxidant-related parameters in the serum and liver tissue, including total superoxide dismutase (SOD), catalase (CAT), glutathione peroxidase (GPx), and malondialdehyde (MDA), were quantified according to the manufacturer’s protocols provided by Beijing Boxbio Science & Technology Co., Ltd. (Beijing, China).

### 2.6. Metabolomic Method

Our metabolomic approach is described in detail in the Supplementary Materials. Briefly, 6 samples were randomly selected from each group for metabolomic analysis. Every 100 mg solid sample was weighed and transferred into a 2 mL centrifuge tube with a grinding bead. The metabolites were extracted using 400 μL of methanol–water solution with an internal standard, followed by grinding, ultrasonic extraction, incubation, and centrifugation. The supernatants were collected for LC-MS/MS analysis. The quality control (QC) sample was prepared by mixing equal volumes of all the samples and tested like the analytic samples were. It represented the whole set and was injected every 6 samples to monitor stability. A pooled QC sample was prepared to monitor the stability of the analysis. LC-MS/MS analysis was conducted on a Thermo UHPLC-Q Exactive HF-X system with specific mobile phases and MS conditions. The mass spectrometric data were collected using a Thermo UHPLC-Q Exactive HF-X Mass Spectrometer (Waltham, MA, USA) with the ESI source in the positive and negative modes. The optimal conditions included a source temperature of 425 °C, sheath gas flow rate of 50 arb, aux gas flow rate of 13 arb, ISVF at −3500 V (negative) and 3500 V (positive), and a normalized collision energy of 20–40–60 V for MS/MS. The full MS resolution was 60,000, the MS/MS resolution was 7500, and data acquisition was performed in DDA mode over a mass range of 70–1050 *m*/*z*.

### 2.7. Statistical Analysis

Biochemical data (means ± SEM) were subjected to one-way ANOVA analysis. Outliers were identified using Grubbs’ test (α = 0.05) and excluded. Data were log-transformed where necessary to meet normality assumptions for ANOVA. Following the verification of the homogeneity of variances, the Tukey multiple range test was conducted for pairwise comparisons. Differences with *p* values less than 0.05 were deemed statistically significant. All graphical representations were generated using GraphPad Prism software version 10.4 (San Diego, CA, USA). Statistical analyses were performed using SPSS version 24.0 (IBM Corporation, Armonk, NY, USA).

The statistical analysis of the metabolomics data is described in the Supplementary Materials. Briefly, the LC/MS raw data were preprocessed using Progenesis QI software version 4.3 (Waters Corporation, Milford, CT, USA), generating a three-dimensional CSV matrix containing sample information, metabolite names, and mass spectral response intensity. The data cleaning steps included removing internal standard peaks, false positives, and redundant peaks. To minimize errors attributable to sample preparation and instrument instability, the intensities of the mass spectrometry peaks were normalized using the sum normalization method, thereby generating a normalized data matrix. The remaining variables were then log10-transformed to construct the final data matrix for subsequent analysis. Metabolites were identified via databases like the Human Metabolome Database (HMDB), Metlin, and Majorbio Database. The cleaned data matrix was uploaded to the Majorbio cloud platform for further analysis. Preprocessing involved retaining metabolic features detected in at least 80% of samples, normalizing data, and excluding variables with RSD > 30%. Principal component analysis (PCA) and partial least squares discriminant analysis (PLS-DA) were performed using the R package “ropls” to identify significantly different metabolites. The differential metabolites were mapped to biochemical pathways using the KEGG (Kyoto Encyclopedia of Genes and Genomes) database for enrichment analysis.

## 3. Results

### 3.1. Effects of TPs Supplementation on Survival Rate

**Figure 1:**
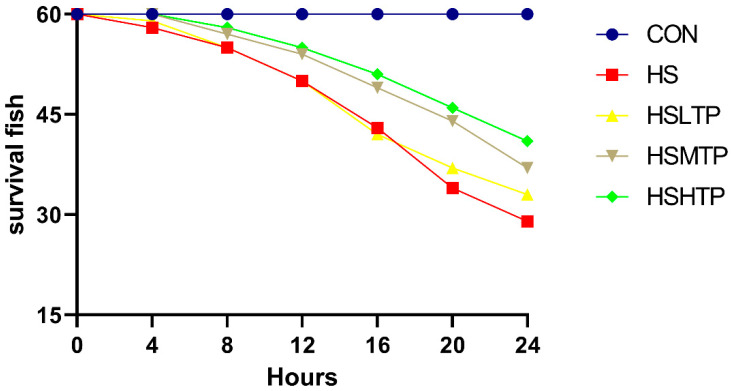
Survival rates of hybrid crucian carp (*n* = 60) fed different levels of tea polyphenols (TPs) under heat-stress exposure.

This experiment employed heating to simulate extreme environmental temperature changes and further investigate the effect of TP supplementation on the survival rate of hybrid crucian carp (HCC2). As shown in Figure 1, under suitable environmental conditions (26 °C), all fish in the CON group survived over 24 h. In contrast, the survival rate in the experimental group without TP supplementation (HS group) was significantly lower. Compared to the HS group (survival rate at 24 h = 48.3%), the survival rates of the HSLTP, HSMTP, and HSHTP groups were 55.0%, 61.6%, and 68.3%, respectively.

### 3.2. Effects of TPs Supplementation on Stress Indicators

**Figure 2:**
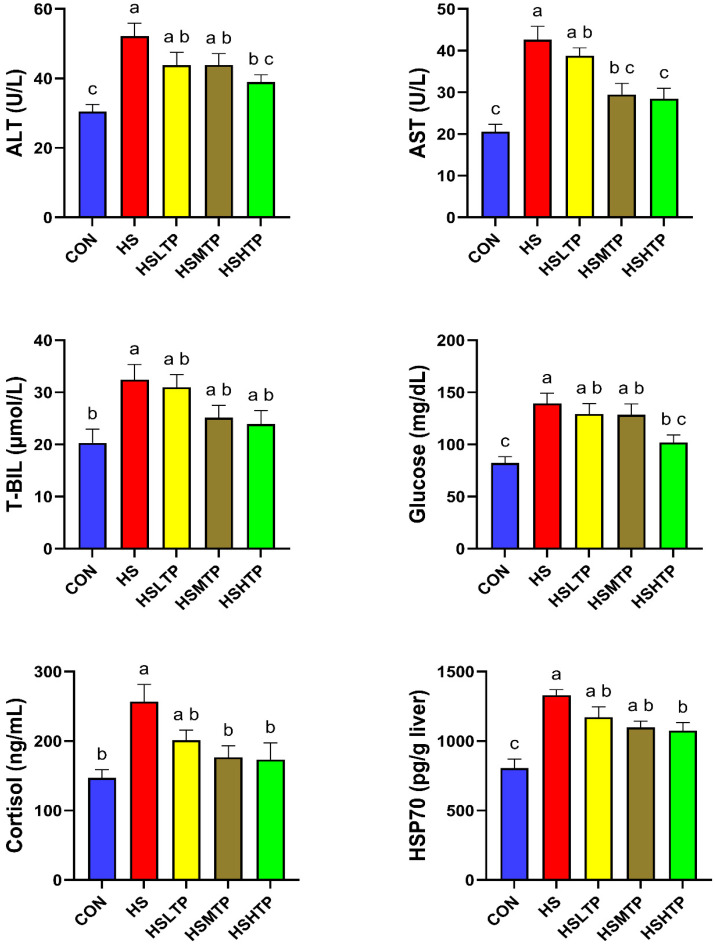
Stress indicators of hybrid crucian carp fed different levels of tea polyphenols (TPs) under heat-stress exposure. Bars with different letters mean significant differences at (*p* < 0.05) by Tukey’s multiple range test. Values expressed as means ± SEM (*n* = 6). Abbreviations: alanine transaminase (ALT), aspartate aminotransferase (AST), total bilirubin (T-BIL), and heat shock protein-70 (HSP-70).

The effects of acute heat stress on the stress indicators of hybrid crucian carp are shown in Figure 2. Acute heat stress significantly increased the serum ALT and AST activity of the hybrid crucian carp; however, it did not affect the serum T-BIL concentration. Dietary TPs significantly decreased the activity of ALT and AST, and the minimum values of ALT and AST appeared in the HSHTP group (*p* < 0.05). In terms of cytokines, hybrid crucian carp in the HSMTP and HSHTP groups displayed lower (*p* < 0.05) cortisol than those in the HS group. Similarly, the fish in the HSHTP group exhibited significantly lower (*p* < 0.05) levels of glucose and HSP70 activity (*p* < 0.05) compared to those in the HS group.

### 3.3. Effects of TPs Supplementation on Antioxidant Capacity

**Figure 3:**
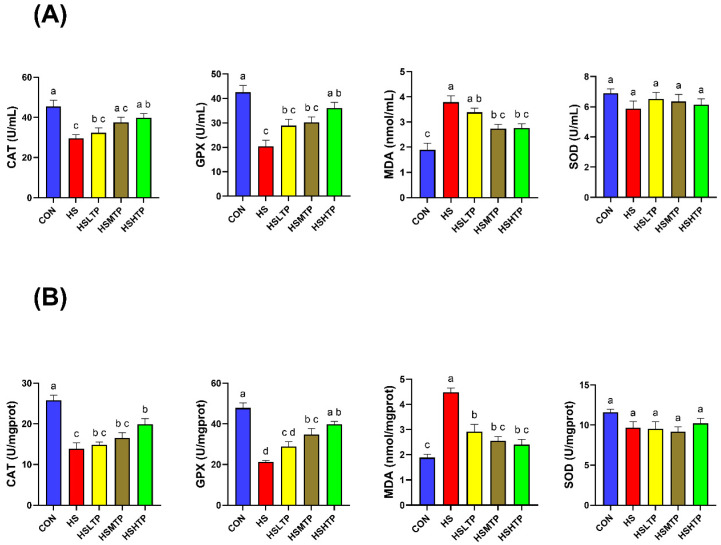
Serum (**A**) and liver (**B**) antioxidant parameters of hybrid crucian carp fed different levels of tea polyphenols (TPs) under heat-stress exposure. Bars with different letters mean significant difference at (*p* < 0.05) by Tukey’s multiple range test. Values expressed as means ± SEM (*n* = 6). Abbreviations: catalase (CAT), glutathione peroxidase (GPx), malondialdehyde (MDA), total superoxide dismutase (SOD).

The effects of acute heat stress and dietary TPs on the antioxidant markers of the hybrid crucian carp are shown in Figure 3. Compared with the CON group, the CAT and GPx enzyme activity of the fish in the HS group were significantly lower (*p* < 0.05), while the levels of MDA were significantly higher (*p* < 0.05) in both the serum and liver. Compared with the fish in the HS group, the HSHTP group exhibited higher (*p* < 0.05) CAT bioactivity; both the HSMTP and HSHTP groups showed higher (*p* < 0.05) GPx enzyme activity in the liver; all the TP-supplemented groups showed decreased (*p* < 0.05) concentrations of MDA in their livers.

### 3.4. Sample Information and Comparative Analysis

A total of 7944 metabolic peaks were detected in the metabolite composition of the samples, comprising 4898 peaks in positive ion mode and 3046 peaks in negative ion mode. After the preprocessing steps (described in Supplementary Material), peaks with CV > 30% in the QCs or present in <80% of the samples were excluded. Blank subtraction removed the contaminants. As a result, 1247 metabolites were identified, including 790 metabolites in positive ion mode and 457 metabolites in negative ion mode. The total ion numbers and identification statistics are shown in Supplementary Table S2.

**Figure 4:**
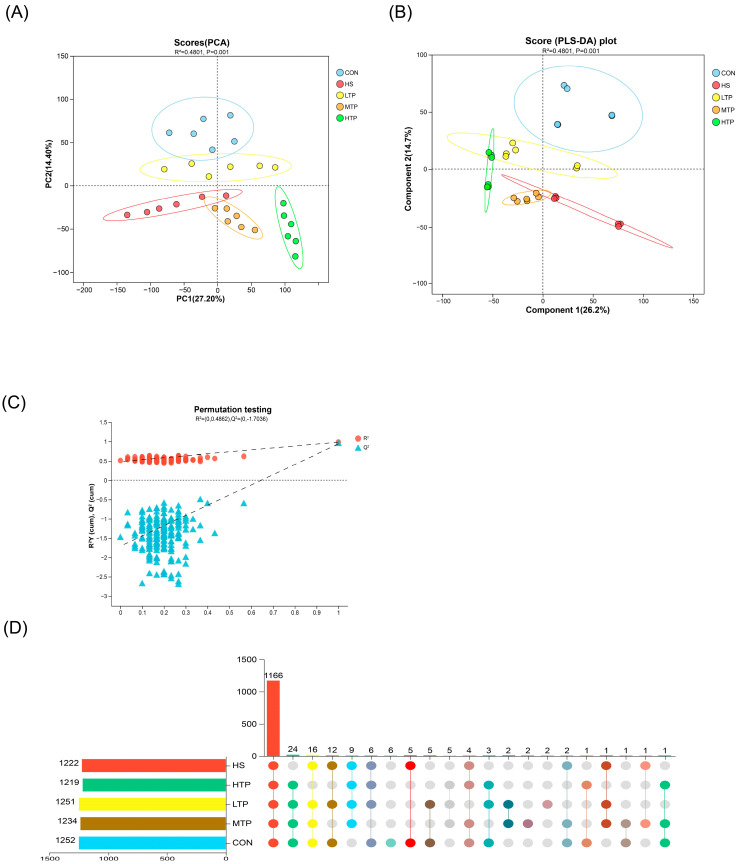
PCA (**A**), PLS-DA (**B**), PLS-DA replacement test (**C**), and Venn Upset (**D**) diagrams of hybrid crucian carp livers in response to heat stress in mixed (both positive and negative) ion mode.

The PCA and PLS-DA score plots (Figure 4A,B) illustrate the metabolic profiles of each group. The verification results of the PLS-DA model are shown in Figure 4C. The variance contribution, as indicated by the R-squared (R^2^) value, represents the extent to which group factors account for the differences among samples. In the PLS-DA, the R^2^Y and Q^2^ factors assessed the fit ability of the model. The control group (CON) and the experimental groups (HSLTP, HSMTP, and HSHTP) changed significantly when compared to the heat stress group (HS) in the mixed (both positive and negative) ion mode. PC1 (R^2^ = 27.2%) and PC2 (R^2^ = 14.4%) represent the first and second principal component contributions, respectively. Tests for differences between the groups were performed using the Adonis method. The *p* values less than 0.05 (specifically, *p* = 0.001 for both Figure 4A,B) indicated statistically significant differences. The verification results (Figure 4C) of the PLS-DA model showed that R^2^ was above Q^2^ in mixed ion mode, and the intercept of the Q^2^ regression line and Y (vertical) axis was −1.7036, indicating that the model was well fitted, predictable, and suitable for subsequent data analysis. The Upset Venn plot (Figure 4D) illustrates the intersections of the differential metabolites (DMs) across various comparisons. The left histogram illustrates the frequency of the DMs detected within each group. The right histogram elucidates the intersections arising from the pairwise comparisons, emphasizing the overlap of DMs across groups. A higher R^2^ value signifies a greater degree of explanation for the variability in group differences.

**Figure 5:**
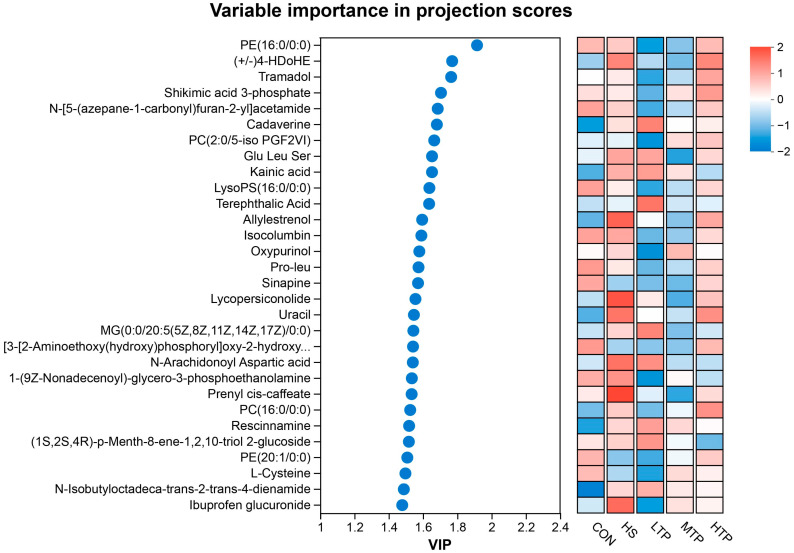
The top 30 variable importance in projection (VIP) scores of metabolites in hybrid crucian carp livers in response to heat stress. The selected metabolites were those with VIP > 1. The heat maps with red or blue boxes on the right indicate high and low abundance ratios, respectively. The VIP scores were based on the PLS-DA model.

In PLS-DA analyses, variable importance in projection (VIP) is employed to evaluate the significance of independent variables in explaining the dependent variable. The VIP scores of metabolites can be used to identify variables that significantly contribute to a model. Typically, a threshold of VIP > 1 is applied to highlight variables with substantial influence on a model’s classification, thereby identifying DMs or potential biomarkers. In Figure 5, the top 10 VIP metabolites were PE(16:0/0:0) (VIP = 1.915), (+/−)4-HDoHE (VIP = 1.770), Tramadol (VIP = 1.764), Shikimic acid 3-phosphate (VIP = 1.705), N-[5-(azepane-1-carbonyl)furan-2-yl]acetamide (VIP = 1.686), Cadaverine (VIP = 1.681), PC(2:0/5-iso PGF2VI) (VIP = 1.665), Glu Leu Ser (VIP = 1.653), Kainic acid (VIP = 1.652), and LysoPS (16:0/0:0) (VIP = 1.637).

### 3.5. Metabolite Annotation and Pathway Analysis

**Figure 6:**
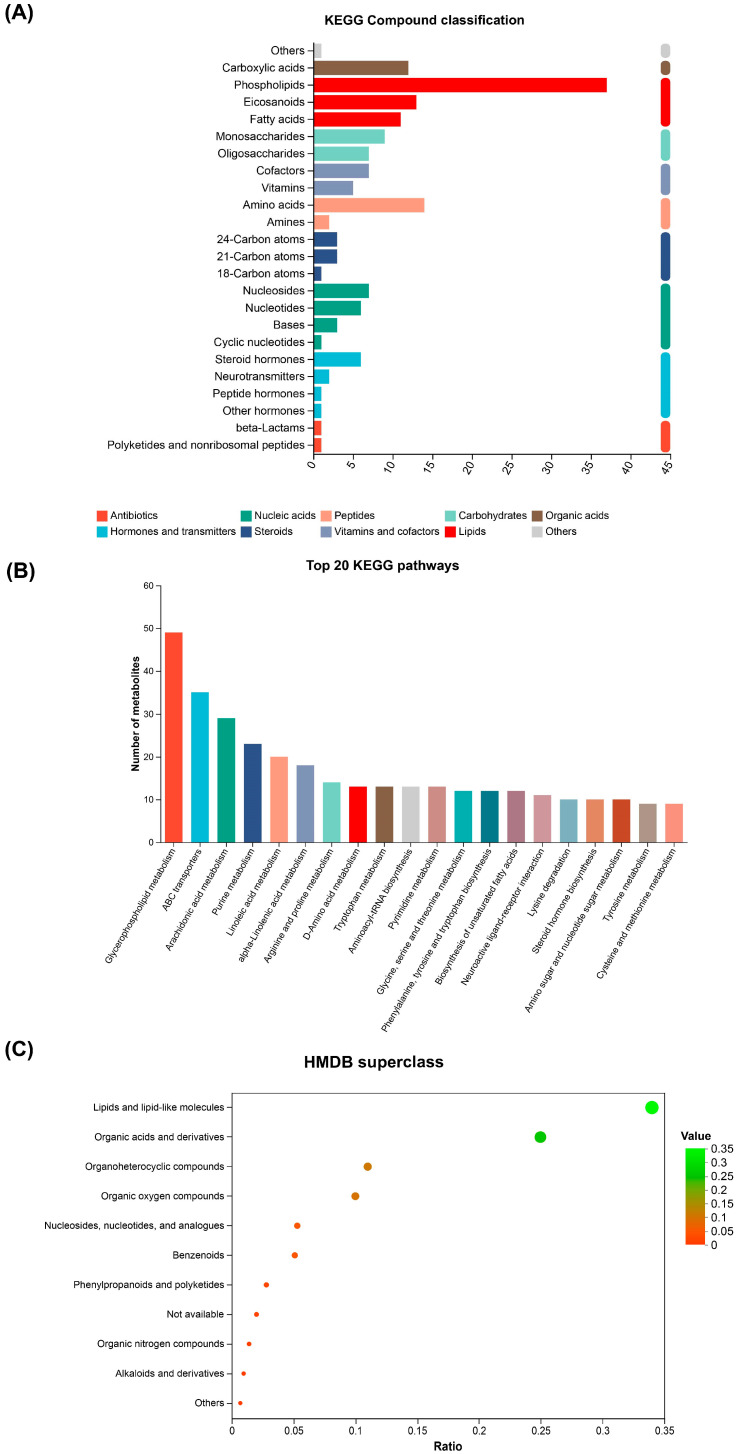
Diagrams of the classification of KEGG compounds (**A**), KEGG pathways (**B**), and HMDB compounds (**C**) of hybrid crucian carp livers in response to heat stress. (**A**) The vertical coordinate is the classification of KEGG compounds and the horizontal coordinate is the number of compounds of this type. The color of the bars indicates that they belong to a primary classification class of the compounds. (**B**) From left to right, the numbers of metabolites contained are ranked from high to low. The higher the column, the more metabolites involved in this pathway among the identified metabolites, and the more active the biological pathway. The Benjamini–Hochberg method was applied to control the false discovery rate (*q* < 0.1). (**C**) The horizontal coordinate is the ratio, which represents the relative proportion of the number of metabolites in each classification; the vertical coordinate is each classification; and the color and size of bubbles represent the ratio values.

According to the annotations from the KEGG, the metabolites were classified into 24 categories of compounds with biological roles (Figure 6A), such as phospholipids, amino acids, eicosanoids, carboxylic acids, fatty acids, monosaccharides, cofactors, oligosaccharides, nucleosides, and nucleotides. To further explore the effects of TPs in mitigating heat stress, we applied the KEGG compound database to enrich these DMs for metabolic pathway analysis (Figure 6B). A total of 69 metabolic pathways were enriched between the five groups. The significant metabolomic pathways included glycerophospholipid metabolism, arachidonic acid metabolism, purine metabolism, linoleic acid metabolism, alpha-linolenic acid metabolism, arginine and proline metabolism, tryptophan metabolism, and D-amino acid metabolism. The majority of these pathways were associated with amino acid and lipid metabolism. To achieve a clearer presentation, we employed a histogram to illustrate the level-two terms of the KEGG pathways (Supplementary Figure S1). A comparable analysis was performed utilizing the HMDB compound database (Figure 6C) to validate the findings obtained from the KEGG database. A total of 393 lipids and lipid-like molecules were identified, representing 34.38% of all the detected compounds. Additionally, 289 organic acids and their derivatives were identified, accounting for 25.28% of the total compounds.

### 3.6. Metabolite Clustering Analysis

**Figure 7:**
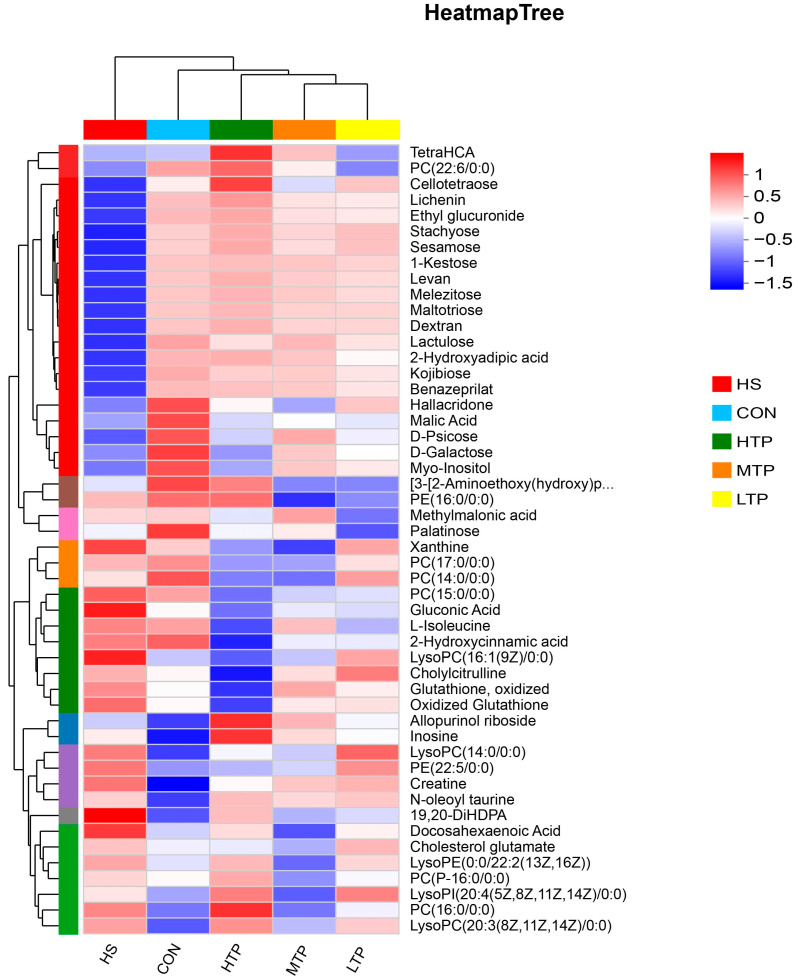
A heatmap of the differentially expressed metabolites in hybrid crucian carp livers in response to heat stress. Each column represents a group of samples and each row represents the average value of a metabolite in that group. The colors in the figure indicate the relative size of metabolite expression in this group of samples. The left side is a tree diagram of the metabolite cluster and the right side contains the metabolite names. At the top is a tree diagram of sample clustering, and the bottom lists the experimental groups’ names.

We performed a cluster analysis of metabolites from each group because metabolites with similar expression patterns are often functionally correlated. The top 30 abundance metabolites are shown in Figure 7 as a heatmap, distinguished from each other through the clustering dendrogram. It is possible to visually see the changing trends of different metabolites in different groups, indicating which metabolites have undergone significant changes. The differences in upregulated and downregulated metabolites between each group are shown in Supplementary Figure S2. Based on the metabolite clustering analysis, dietary TPs protect hybrid crucian carp from acute heat stress mainly by modulating lipid metabolism in the liver.

## 4. Discussion

The escalating challenges posed by global warming to aquaculture necessitate innovative nutritional strategies to enhance thermal resilience in economically important species. Our study provides compelling evidence that dietary tea polyphenols (TPs) confer significant protection against acute heat stress in hybrid crucian carp HCC2 through multifaceted mechanisms involving antioxidant enhancement and lipid metabolic regulation. As a traditional Chinese medicine, green tea, renowned for its heat-clearing properties, is commonly employed to prevent and alleviate heat-related illnesses induced by elevated ambient temperatures [24]. In certain circumstances, aquatic animals can adapt to stress induced by changes in their surrounding environment. However, if this stress exceeds a specific threshold, physiological activity may begin to decline, even leading to death [25]. Survival rate is an important index to evaluate heat-stress injury, Dawood et al. reported that the survival rate of Nile tilapia was less than 60% when exposed to 48 h of heat stress at 36 °C [26]. In this study, the supplementation of TPs was shown to enhance fish tolerance to drastic fluctuations in high temperature, in agreement with Duong et al. who found that green tea extract (GTE) enhanced the survival of abalone (*Haliotis laevigata* Donovan) cultured in high water temperatures [27]. While survival improved by 20%, combining TPs with cooling protocols or probiotics may enhance their efficacy during prolonged heatwaves.

Heat stress (HS) has been shown to adversely affect fish livers and can lead to extensive liver injury [28]. ALT and AST are critical indicators of hepatic diseases in fish. Normally, they are not released into the blood. Elevated serum levels of ALT and AST indicate increased membrane permeability and hepatocyte damage [29]. These cause further metabolic disorders, which in turn lead to the accumulation of T-BIL. Khieokhajonkhet et al. found through a ten-week chronic heat stress experiment on goldfish that, as the temperature rose, the levels of ALT and AST in the fish serum showed a linear upward trend, indicating that liver function might have been significantly impaired [30]. However, in the present study, TP supplementation demonstrated a hepatoprotective effect, as reflected by the decreased levels of ALT and AST. Similarly, Lin et al. found that adding dietary TPs to an oxidized fish oil diet enhanced the liver health of spotted seabass (*Lateolabrax maculatus*) [20]. Moreover, blood cortisol and glucose levels are key indicators of the physiological response to heat stress in fish, as elevated blood glucose levels result from increased cortisol concentrations promoting gluconeogenesis in the liver [31]. In this study, fish fed with TPs showed lower cortisol and glucose levels during heat stress compared to the HS group. These results suggested TPs have an anti-stress effect by inhibiting cortisol elevation. We speculated that EGCG exerted restorative effects on blood cortisol levels stimulated under stress-induced neural injuries in rats [32]. In the current study, HSP70 was characterized as a significant tool for promoting cell survival by protecting cells from stress. The relative expression of HSP70 serves as a potential stress marker in aquatic animals, providing protection and thermotolerance during heat stress [33]. Our study found that hybrid crucian carp fed TPs had reduced HSP70 levels in serum, suggesting a mitigated effect of heat stress. Lower HSP70 levels in the TP groups indicates reduced proteotoxic stress, not impaired chaperone function, as survival and antioxidant markers improved. Similarly, a previous investigation showed that EGCG mitigates heat-stress-induced fat deposition by targeting HSP70 in porcine subcutaneous preadipocytes [34].

Reactive oxygen species (ROS) are highly oxidizing compounds, ions, and free radicals. The body relies on its antioxidant system to maintain health. External factors like temperature and salinity can cause abnormal metabolism, leading to excessive ROS production [35]. To avoid damage from ROS, the antioxidant defense system in fish eliminates reactive oxygen species produced by cell metabolism, maintaining oxidation–antioxidant equilibrium. The antioxidant system of aquatic animals primarily includes enzymes like CAT and SOD [36]. In addition, the overproduction of ROS can lead to increased lipid peroxidation, resulting in elevated MDA levels. The persistent presence of MDA can damage cellular components by breaking down DNA, proteins, and cytoplasmic structures [37]. The reduction of lipid peroxides is catalyzed by GPx, which utilizes NADPH to reduce glutathione disulfide (GSSG) into two glutathione (GSH) molecules [38]. While MDA is a common peroxidation marker, future work should assess 4-Hydroxynonenal (4-HNE) adducts for comprehensive lipid damage evaluation. The findings of this study demonstrated that heat stress increased MDA content and decreased CAT and Gpx activities in hybrid crucian carp, suggesting that elevated temperatures induced oxidative stress. In our study, pretreatment with TPs mitigated heat-stress-induced ROS generation and MDA accumulation by restoring antioxidant enzyme activities. These results were in line with studies of other carp fishes, such as juvenile common carp (*Cprinus carpio*) [39] and gibel carp (*Carassius gibelio*) [40]. As renowned antioxidants, TPs can directly engage in redox reactions within fish and positively influence their antioxidant systems [41]. TPs likely exert dual antioxidant effects: (1) direct ROS scavenging via catechol moieties and (2) the Nrf2-mediated upregulation of SOD and CAT, as evidenced in heat-stressed poultry [42]. However, the molecular mechanisms by which tea polyphenols influence oxidative stress in fish remain unclear. Future studies should quantify Nrf2 activation in TP-supplemented fish.

The liver is one of the most critical metabolic organs in fish, exhibiting a significant capacity for substance metabolism and performing essential physiological functions including detoxification, immune defense, and hormone synthesis. It plays a vital role in mitigating the effects of heat stress and safeguarding organisms from damage caused by elevated temperatures [43]. Numerous studies have demonstrated that acute heat stress can significantly alter liver-associated metabolic pathways. Bao et al. found that the exposure of tilapia to 35 °C for 48 h resulted in the notable upregulation of miR-1 and miR-122 expression in the liver [44]. Therefore, we designated the liver as the primary target organ for our metabolomics analysis. Metabolomics enables the capture of dynamic changes in small-molecule metabolites and provides a systematic evaluation of alterations in these metabolites within biological systems following stimulation or perturbation. This approach facilitates the investigation of the relationship between metabolic pathways and biological functions [45].

Heat stress is a significant environmental challenge that affects cellular homeostasis and metabolic processes in various organisms. Among the critical metabolic pathways influenced by heat stress are glycerophospholipid and arachidonic acid metabolism [46]. These pathways play essential roles in maintaining membrane integrity, signaling, and inflammatory responses, all of which are crucial for cellular adaptation to elevated temperatures. In this study, we utilized metabolomics to analyze the liver of HCC2 carp and found that heat stress impacted lipid metabolism. Our metabolomic profiling reveals two pivotal regulatory axes in TP-mediated heat adaptation: (1) Glycerophospholipid remodeling: The 34.38% lipid/lipid-like molecules identified (Figure 6C) predominantly participate in membrane structural adaptation. Thermal stress typically induces membrane fluidity alterations through lipid peroxidation [41], but TP supplementation appears to maintain phospholipid composition critical for cellular compartmentalization and signal transduction. Elevated phosphatidylcholines (PCs, 16:0/18:1) in TP groups (Figure 6) may stabilize membranes by increasing acyl chain saturation, as shown in heat-tolerant yeast [47]. (2) Eicosanoid modulation: The enrichment of arachidonic acid metabolism pathways (Figure 6B) implies the TP-mediated regulation of inflammatory mediators. While heat stress typically elevates pro-inflammatory prostaglandins [28], TPs may attenuate inflammatory signaling, potentially via COX2 inhibition, though direct evidence requires future cytokine profiling. Suppressed arachidonic acid metabolism (Figure 6B) aligns with reduced cortisol/glucose, suggesting that TPs attenuate stress-induced eicosanoid signaling. Further, TPs may inhibit COX-2 activity, reducing prostaglandin E2 (PGE2) synthesis from arachidonic acid, as demonstrated in murine macrophages [48]. Additionally, phospholipid remodeling enzymes, such as lysophosphatidylcholine acyltransferase (LPCAT), could be upregulated by TPs to enhance membrane integrity under heat stress [49]. Yan et al. found that largemouth bass exposed to 33 °C for 8 weeks had a poorer growth performance, more severe liver damage, and altered lipid levels [50]. The expression of genes related to gluconeogenesis and lipid metabolism was downregulated, while glycolysis-related genes were upregulated at 33 °C. This suggests that enhanced glycolysis and inhibited gluconeogenesis and lipid metabolism may improve the capacity to cope with heat stress.

While demonstrating clear benefits, our experimental design presents certain limitations. The 24 h acute stress model mimics short-term heatwaves, which are increasingly frequent due to climate change. Chronic heat stress (weeks to months) may induce distinct adaptations, such as the sustained upregulation of heat shock proteins or shifts in energy metabolism, warranting further investigation. Furthermore, the precise molecular intermediaries linking lipid metabolic shifts to enhanced thermal tolerance warrant investigation through targeted lipidomic and transcriptomic approaches.

## 5. Conclusions

The present study established dietary tea polyphenols as an effective, eco-friendly intervention against acute heat stress in hybrid crucian carp HCC2. TP supplementation modulated key metabolic pathways in the liver, particularly those associated with lipid metabolism, which are crucial for coping with heat-induced physiological challenges. These findings provide a scientific basis for implementing TP-supplemented feeding regimes in warm-water aquaculture systems. Our metabolomics approach identifies lipid metabolites as potential biomarkers for heat stress mitigation. Future research directions should explore genetic biomarkers of TP responsiveness, long-term impacts on growth performance, and the economic feasibility of large-scale TP incorporation in aquafeeds. Based on dose–response trends and cost–benefit analysis, 200–400 mg/kg TPs are recommended for aquaculture applications.

## Figures and Tables

**Table 1:** Formulation and proximate composition of experimental diets ^a^ (%,dry matter).

Ingredients	CON	HS	HSLTP	HSMTP	HSHTP
Wheat Flour	29.7	29.7	29.69	29.68	29.66
Soybean Meal	27	27	27	27	27
Rapeseed Meal	15	15	15	15	15
Peanut Hulls	12	12	12	12	12
Fish Meal	10	10	10	10	10
Fish Oil	2	2	2	2	2
Ca(H_2_PO_4_)_2_	1.5	1.5	1.5	1.5	1.5
Soybean Oil	1	1	1	1	1
Additive Premixes ^b^	1	1	1	1	1
Choline Chloride	0.5	0.5	0.5	0.5	0.5
Antimicrobial Agents	0.2	0.2	0.2	0.2	0.2
Antioxidants	0.1	0.1	0.1	0.1	0.1
Tea Polyphenols ^c^	0	0	0.01	0.02	0.04
Proximate Composition (%)					
Crude Protein	35.01	35.01	35.01	35.01	35.00
Crude Fat	5.38	5.38	5.38	5.38	5.38
Moisture	6.09	6.09	6.09	6.09	6.09

^a^ The CON and HS groups were fed a basal diet without tea polyphenols; HSLTP, HSMTP, and HSHTP groups were fed a basal diet with 100, 200, and 400 mg/kg tea polyphenols. ^b^ The vitamin premix and mineral premix provided the following per kg of feed: VA, 10,800 IU; VD3, 4000 IU; VE, 40 IU; VK3, 3.4 mg; VB1, 1.6 mg; VB2, 12 mg; VB6, 6 mg; VB12, 0.05 mg; biotin, 0.2 mg; folic acid, 2 mg; niacin, 50 mg; D-Calcium, 25 mg; Fe, 80 mg; Cu, 100 mg; Mn, 50 mg; Zn, 90 mg; Co, 1 mg; Se, 0.17 mg; I, 0.15 mg. ^c^ The tea polyphenols were provided by the National Research Center of Engineering Technology for Utilization of Functional Ingredients from Botanicals, Hunan Agricultural University, Changsha, China.

## Data Availability

The data presented in this study are available on request from the corresponding author due to privacy.

## Supplementary Materials

The following supporting information can be downloaded at: https://www.mdpi.com/article/10.3390/metabo15040229/s1, Section S1: The detailed metabolomic method. Table S1: The certification of the tea polyphenols. Table S2: Total ion numbers and identification statistics. Figure S1: Histogram of level-2 terms of KEGG metabolic pathways. Figure S2: Bar chart of differential metabolite expression.
