# Supplementary material for: Dietary Tea Polyphenols Alleviate Acute-Heat-Stress-Induced Death of Hybrid Crucian Carp HCC2: Involvement of Modified Lipid Metabolisms in Liver
Source: Metabolites. 2025 Mar 27;15(4):229. doi: 10.3390/metabo15040229 (PMC12028923; doi:10.3390/metabo15040229)
Supplement: Supplementary file 1 [file metabolites-15-00229-s001.zip › metabolites-3493660-supplementary.pdf]

S1.Metabolomic method

S1.1. Metabolite Extraction

A 100 mg liver sample was weighed and transferred into a 2 mL centrifuge tube, followed by the addition of a 6 mm diameter grinding bead. For metabolite extraction, 400 μL of extraction solution (methanol: water = 4:1, v/v) containing 0.02 mg/mL of internal standard (L-2-chlorophenylalanine) was added. The samples were subsequently ground using the Wonbio-96c frozen tissue grinder (Shanghai Wanbo Biotechnology Co., Ltd.) for 6 minutes at -10°C and 50 Hz. This was followed by low-temperature ultrasonic extraction for 30 minutes at 5°C and 40 kHz. The samples were then incubated at -20°C for 30 minutes, followed by centrifugation at 4°C for 15 minutes at 13,000 g. The supernatant was carefully collected and transferred to an injection vial for LC-MS/MS analysis.

S1.2. Quality control sample

As a part of the system conditioning and quality control process, a pooled quality control sample (QC) was prepared by mixing equal volumes of all samples. The QC samples were disposed of and tested in the same manner as the analytic samples. It helped to represent the whole sample set, which would be injected at regular intervals (every 6 samples) to monitor the stability of the analysis.

S1.3. UHPLC-MS/MS) analysis

The LC-MS/MS analysis of the sample was conducted on a Thermo UHPLC-Q Exactive HF-X system equipped with an ACQUITY HSS T3 column (100 mm × 2.1 mm i.d., 1.8 μm; Waters, USA) at Majorbio Bio-Pharm Technology Co. Ltd. (Shanghai, China). The mobile phases consisted of 0.1% formic acid in water: acetonitrile (95:5, v/v) (solvent A) and 0.1% formic acid in acetonitrile: isopropanol: water (47.5:47.5, v/v) (solvent B). The flow rate was 0.40 mL/min and the column temperature was 40℃.

MS conditions: The mass spectrometric data were collected using a Thermo UHPLC-Q Exactive HF-X Mass Spectrometer equipped with an electrospray ionization (ESI) source operating in positive mode and negative mode. The optimal conditions were set as follows: source temperature at 425℃; sheath gas flow rate at 50 arb; Aux gas flow rate at 13 arb; ion-spray voltage floating (ISVF) at -3500V in negative mode

and 3500V in positive mode, respectively; Normalized collision energy, 20-40-60V rolling for MS/MS. Full MS resolution was 60000, and MS/MS resolution was 7500. Data acquisition was performed with the Data Dependent Acquisition (DDA) mode. The detection was carried out over a mass range of 70-1050 m/z.

S1.4. Data analysis

The pretreatment of LC/MS raw data was performed by Progenesis QI (Waters Corporation， Milford, USA) software, and a three-dimensional data matrix in CSV format was exported. The information in this three-dimensional matrix included: sample information, metabolite name, and mass spectral response intensity. Internal standard peaks, as well as any known false positive peaks (including noise, column bleed, and derivatized reagent peaks), were removed from the data matrix, redundant, and peak pooled. At the same time, the metabolites were identified by searching databases, and the main databases were the HMDB (http://www.hmdb.ca/), Metlin (https://metlin.scripps.edu/), and the Majorbio Database.

The data matrix obtained by searching the database was uploaded to the Majorbio cloud platform (https://cloud.majorbio.com) for data analysis. First, the data matrix was pre-processed, as follows: At least 80% of the metabolic features detected in any set of samples were retained. After filtering, for specific samples with metabolite levels below the lower limit of quantification, the minimum metabolite value was estimated and each metabolic signature was normalized to the sum. To reduce the errors caused by sample preparation and instrument instability, the response intensities of the sample mass spectrometry peaks were normalized using the sum normalization method, to obtain the normalized data matrix. Meanwhile, the variables of QC samples with relative standard deviation (RSD) > 30% were excluded and log10 logarithmic zed, to obtain the final data matrix for subsequent analysis.

Then, the R package "ropls"(Version 1.6.2) was used to perform principal component analysis (PCA) partial least squares discriminant analysis (PLS-DA), and 7-cycle interactive validation evaluating the stability of the model. The metabolites with VIP>1, $p<0.05$ were determined as significantly different metabolites based on the Variable importance in the projection (VIP) obtained by the OPLS-DA model and the

p-value generated by the student's t-test.

Differential metabolites among the two groups were mapped into their biochemical pathways through metabolic enrichment and pathway analysis based on the KEGG database (http://www.genome.jp/kegg/). These metabolites could be classified according to the pathways they are involved in or the functions they perform. Enrichment analysis was used to analyze a group of metabolites in a function node whether appears or not. The principle was that the annotation analysis of a single metabolite develops into an annotation analysis of a group of metabolites. Python package "scipy.stats" (https://docs.scipy.org/doc/scipy/) was used to perform enrichment analysis to obtain the most relevant biological pathways for experimental treatments.

S2.Supplementary tables

S2.1. Table S1

Tea polyphenols (TPs) were provided by the National Research Center of Engineering Technology for Utilization of Functional Ingredients from Botanicals, Hunan Agricultural University, Changsha, China. The certification for TPs is shown in Table S1.

Table S1 The certification of tea polyphenols.

Examination Items	Unit	Specification	Result
Appearance		Brown powder	Confirmed
Total Tea Polyphenols	%	>= 98.0%	98.50%
Total Catechins	%	>= 80.0%	82.20%
Epigallocatechin-3-gallate (EGCG)	%	>= 50.0%	52.20%
Caffeine	%	<=2.0%	0.80%
Water Content	%	<=5.0%	3.20%
Ash	%	<=1.0%	0.50%
Granularity		>= 95.0% through 80 Mesh	Confirmed
Lead	PPM	<=5.0	Confirmed
As	PPM	<=2.0	Confirmed
Total Bacterial	cfu/g	<=1000	Confirmed
Mold & Yeast	cfu/g	<=100	Confirmed
Colibacillus		Negative	Negative
Salmonella		Negative	Negative

S2.2. Table S2

The matrix files identified through the laboratory database search need to undergo data preprocessing steps such as filtering, imputation, normalization, and logarithmic transformation to eliminate or reduce errors introduced during the experiment and analysis process. Before data preprocessing, the column is named "raw", with 801 identifications in positive ion mode and 467 in negative ion mode. After preprocessing, the column is named "origin", and the number of identifications in positive ion mode is 790, while in negative ion mode, it is 457.

Table S2 Total ion numbers and identification statistics

Ion mode	All peaks	Identified metabolites	Metabolites in Library	Metabolites in KEGG
Pos	4898	790	713	409
Neg	3046	457	442	255
Mix	7944	1247	1155	664

(1) Ion mode: The ion mode of the mass spectrometer to detect the substance, mainly: pos (positive ion mode), neg (negative ion mode), and mix (both positive and negative ion mode); (2) All peaks: the number of mass spectrum peaks extracted by software; (3) Identified metabolites: the number of metabolites ultimately identified through primary and secondary mass spectrometry data and database search (self-built database, Metlin, HMDB, etc.); (4) Metabolites in library: the number of metabolites annotated to public databases such as HMDB and Lipidmaps; (5) Metabolites in kegg: The number of metabolites annotated to the KEGG database.

S3. Supplementary figures

S3.1 Figure S1

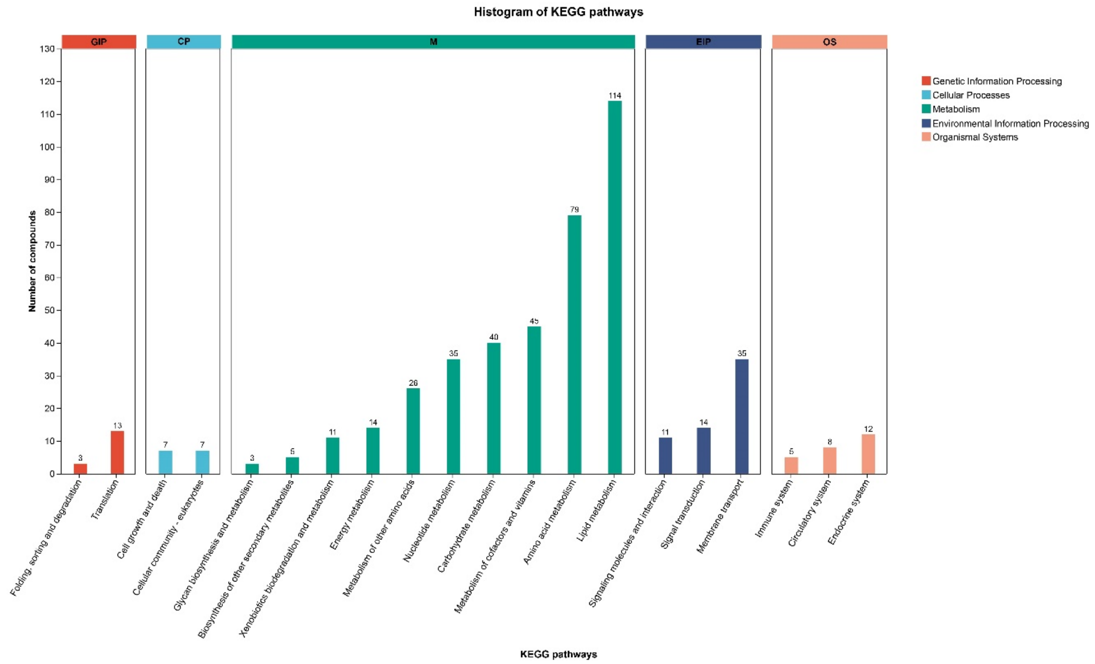

Figure S1. The histogram on level-2 terms of KEGG metabolic pathways

The horizontal axis represents the secondary classification of KEGG metabolic pathways, and the vertical axis represents the number of compounds annotated to the pathway. Different colors for different classifications indicate different categories of metabolic pathways.

S3.2 Figure S2

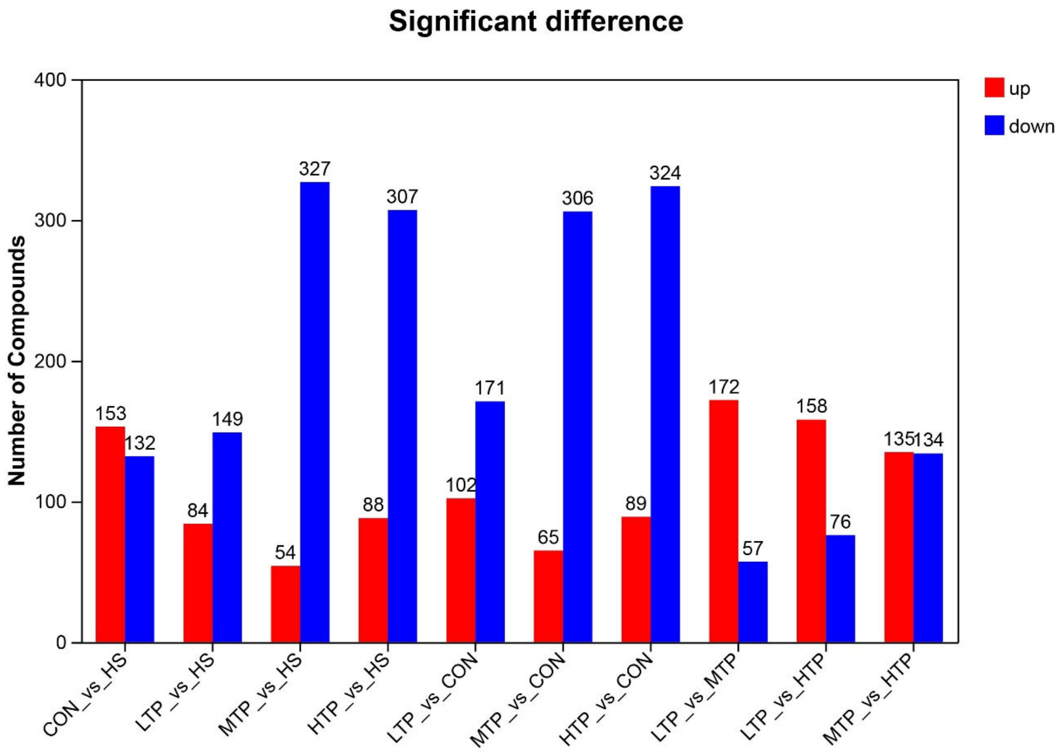

Figure S2. The bar chart of expression difference metabolites

The horizontal axis represents different comparison groups; the vertical axis represents the number of metabolites; in the figure, red indicates up-regulated differential metabolites, and blue indicates down-regulated differential metabolites.
